# Intracellular Eukaryotic Parasites Have a Distinct Unfolded Protein Response

**DOI:** 10.1371/journal.pone.0019118

**Published:** 2011-04-29

**Authors:** Sara J. C. Gosline, Mirna Nascimento, Laura-Isobel McCall, Dan Zilberstein, David Y. Thomas, Greg Matlashewski, Michael Hallett

**Affiliations:** 1 McGill Centre for Bioinformatics, McGill University, Montréal, Québec, Canada; 2 McGill School of Computer Science, McGill University, Montréal, Québec, Canada; 3 Rosalind and Morris Goodman Cancer Centre, McGill University, Montréal, Québec, Canada; 4 Department of Microbiology and Immunology, McGill University, Montréal, Québec, Canada; 5 Faculty of Biology, Technion-Israel Institute of Technology, Haifa, Israel; 6 Department of Biochemistry, McGill University, Montréal, Québec, Canada; University of Bern, Switzerland

## Abstract

Insult to the endoplasmic reticulum (ER) activates the Unfolded Protein Response (UPR), a set of signaling pathways that protect the cell from the potential damage caused by improperly folded proteins. Accumulation of misfolded proteins in the ER lumen initiates a series of signal transduction events via activation of three transmembrane ER proteins: Ire1, Atf6 and PERK. Activation of these proteins results in the transcriptional up-regulation of the components of the folding, trafficking and degradation machinery in the ER. PERK further reduces the load on the ER via the phosphorylation of eIF2α, attenuating general protein translation. It is believed that the UPR evolved as a transcriptional response that up-regulates protein folding machinery in the ER and later gained the ability to decrease ER load by attenuating general protein translation in metazoa. However, our *in silico* analyses of protozoan parasites revealed an absence of proteins involved in the transcriptionally mediated UPR and the presence of both PERK and its target eIF2α. Consistent with these observations, stimulation of the UPR in *Leishmania donovani* identified an absence of up-regulation of the ER chaperone BiP, the canonical ER chaperone modulated by the UPR in higher eukaryotes, while exhibiting increased phosphorylation of eIF2α which has been shown to attenuate protein translation. We further observed that *L. donovani* is more sensitive to UPR inducing agents than host macrophages, suggesting that the less evolved stress response could provide a new avenue for therapeutic treatment of parasitic infections.

## Introduction

**Figure 1:**
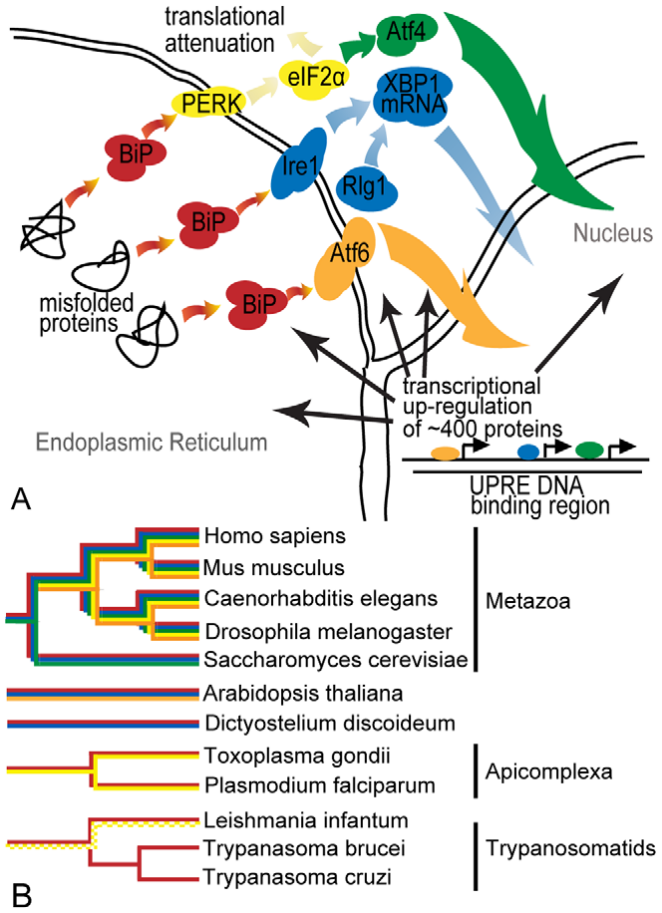
The Unfolded Protein Response across eukaryotes prior to this study. A) The accumulation of unfolded proteins (left) causes BiP (in red) to disassociate from the three transmembrane proteins leading to their subsequent activation: activation of Ire1 (blue) leads to the splicing of XBP1 (or HAC1 in yeast) mRNA; activation of PERK (yellow) results in the phosphorylation of eIF2α which in turn attenuates general cellular translation and up-regulates Atf4 (green); activation of Atf6 (orange) causes it to travel first to the Golgi where it is cleaved and then to the nucleus. Xbp1, Atf4 and Atf6 enter the nucleus where they bind to specific UPR Element (UPRE) binding motifs and up-regulate hundreds of proteins such as BiP. B) Phylogenetic tree of the 12 organisms in this study. Colors of the branches indicate that the proteins with the same color in (A) are present in that organism. Checkered branches indicate results determined by this study.

The Unfolded Protein Response (UPR) is a set of signaling pathways that protect the cell from stress imposed on the endoplasmic reticulum (ER). In metazoa, the accumulation of misfolded proteins in the ER causes the chaperone BiP to disassociate from and subsequently activate three signal transducers: Ire1, PERK and Atf6. Figure 1A shows the signaling pathways initiated by each protein. Inositol Requiring 1 (Ire1α and Ire1β) is a transmembrane kinase/ribonuclease that induces the non-conventional splicing of X box Binding Protein 1 (XBP1, HAC1 in yeast) mRNA. This splicing increases the amount of Xbp1p transcription factor which leads to the up-regulation of protein chaperones, most notably BiP and Protein Disulfide Isomerase (PDI) [1]. PRKR-like Endoplasmic Reticulum Kinase (PERK) phosphorylates the α subunit of eIF2, which causes global translation attenuation by preventing the formation of the 80S complex at the AUG initiator codon [2]. Phosphorylated eIF2α selectively increases the translation of Atf4, a basic-leucine zipper (bZIP) transcription factor that up-regulates ER-resident chaperones [1], [2]. Activating Transcription Factor 6-like proteins (Atf6α, Atf6β, CREB3L2) transcriptionally initiate a gene expression program that includes cell cycle arrest [3]. Together, the inhibition of protein synthesis (by PERK activation) combined with the increase in ER chaperone production (including that of BiP) decrease the accumulation of unfolded proteins in the ER. While all UPR pathways have been implicated in many diseases [4], , the individual pathways have been shown to act independently when faced with varying kinds of stress [6], [7].

Knowing how pathways have evolved can provide valuable insight into their individual function. However, the evolution of the UPR, depicted as a dendrogram in Figure 1B, is not straightforward. The assumption that the Ire1 pathway is the most ancient of the UPR [8], [9] conflicts with evidence of Atf6 in plant [10] and PERK in Apicomplexa [11]. Also, the apparent absence of a transcriptional response in *Giardia lamblia* [12] and *Trypanosoma brucei* [13] suggests that the transcriptional response may be absent in many protozoan parasites, though *T. brucei* is able to mount a UPR-like response through an organism-specific form of mRNA regulation when treated with very high amounts of UPR-inducing chemicals [14]. Though PERK has been identified in *T. brucei* [15], it was found to reside outside the ER in the flagellar pocket. Thus, despite the sequence similarity this protein is unable to act as a functional ortholog because it cannot sense ER stress. While there is evidence of a PERK-based translational UPR in *Toxoplasma gondii* [11], the apparent absence of this pathway in *T. brucei* makes it difficult to assume that this pathway exists in other protozoan parasites.

In this study, we developed a computational model to characterize the UPR across eukaryotes that is able to identify a PERK associated pathway and confirm the absence of a UPR transcriptional response in some protozoa. We validated this model by *in vivo* induction of the UPR in cultured *Leishmania donovani* (*L. donovani*) to measure BiP chaperone levels and eIF2α phosphorylation during experimentally-induced ER stress. We further examined whether *L*. *donovani* was more sensitive to ER stress than host macrophages. Our results suggest *L. donovani* possess a translationally mediated UPR pathway but no change in UPR-specific protein expression, making it more sensitive to ER stress-inducing drugs than its host. The computational model indicates that a transcriptionally-mediated UPR may be absent across parasitic protozoa, suggesting that ER stress could be a therapeutic target in these organisms. Furthermore, our approach can be used to identify other cyto-protective metazoan pathways in an effort to identify new therapeutic targets in parasitic infections.

## Results

### Domain analysis shows absence of UPR-specific transcriptional machinery

**Figure 2:**
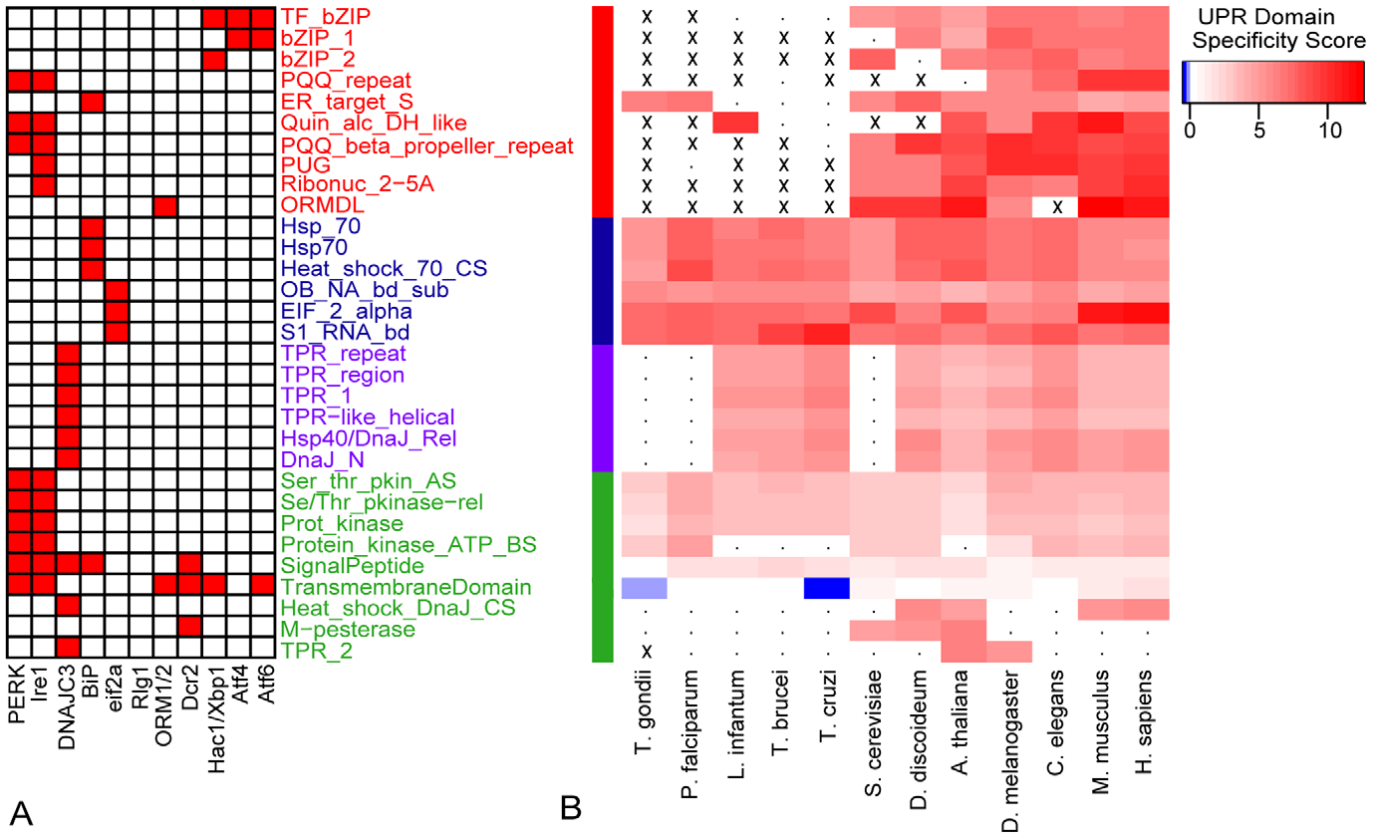
The evolution of functional domains in the UPR. A) UPR proteins in this study (x-axis) and the protein domains that are present in each (y-axis) B) Clustering of the domain specificity scores. The colors of the heatmap represent the UPR domain specificity score of each domain in each species (see Methods). Entries marked with an ‘x’ indicate that the domain is not present in any protein of the species. Entries with a ‘.’ indicate that the domain only appears in proteins that are not implicated in the UPR. The colors on the clusters were assigned to the four clusters with the shortest Euclidean distance between vectors of domain specificity.

The use of standard ortholog detection tools (see Materials and Methods) was unable to identify many important UPR proteins in protozoan parasites including those required for transcriptional regulation of UPR targets (shown in Tables 1 and S1). To investigate whether the transcriptional machinery existed in a highly diverged form (e.g. metazoan Xbp1 and yeast Hac1[16]), we developed a computational model that relies on conservation of protein domains instead of entire amino acid sequences. We collected domains from all proteins involved in the UPR across the 12 eukaryotes in this study. Figure 2A shows the UPR proteins and their respective domains (collected from Interpro [17]). We calculated the specificity value (see Methods) to measure how frequently each domain occurs in UPR proteins relative to non-UPR proteins. Hierarchical clustering of these values shown in Figure 2B reveals clusters of protein domains that have evolved similarly across eukaryotes, indicated by the colors of each cluster of domains.

**Table 1:** UPR proteins across eukaryotes.

Protein Family	UPR Role	HS	MM	DM	CE	SC	AT	DD	TG	PF	LI	TB	TC
BiP	ER chaperone	1	1	1	1	1	3	1	1	1	1	1	1
Ire1	Signal transducer	2	2	2	2	1	2	1	0	0	0	0	0
HAC1/XBP1	Transcription factor	1	1	1	1	1	0	0	0	0	0	0	0
Rlg1	Ligates HAC1 mRNA	0	0	0	0	1	0	0	0	0	0	0	0
Dcr2	Regulates Ire1	0	0	0	0	1	4	1	0	0	0	0	0
PERK	Signal transducer	1	1	1	1	0	0	0	**1**	**1**	**1**	0	0
Eif2α	Attenuates translation	1	1	1	1	1	1	1	1	1	1	1	1
Atf4	Transcription factor	1	1	1	1	1	0	0	0	0	0	0	0
DnaJC3	Regulates PERK/Eif2α	1	1	1	1	0	1	1	0	1	1	1	1
Atf6	Transcription factor	2	2	1	1	0	**1**	0	0	0	0	0	0

Table 1 caption: Protein families involved in the unfolded protein response and their respective size in the eukaryotes examined. Numbers in boldface indicate that the protein is not identifiable via standard sequence homology search but was identified either in the literature [10], [11], or by using techniques described in this manuscript.

The blue and purple clusters include domains that occur within well-conserved proteins such as BiP, eIF2α and DNAJC3 while the green cluster contains domains that have lower specificity values as they reside within a diverse array of proteins across the cell. The red cluster is enriched in protein domains that are present in all species but absent in the protozoan parasites. Surprisingly, these domains are specifically those required in the UPR transcriptional response: the ribonuclease and PUG domains found exclusively within Ire1 and the bZIP domains found in XBP1, Atf6 and Atf4 transcription factors. At least one of these domains is required for proper transcriptional binding to initiate the canonical UPR transcriptional response. While sporadic absence of some domains is expected across such a diverse set of species, the wholesale absence of functionally similar domains suggests that the entire pathway could be absent in protozoan parasites.

### Naive Bayes' classifier identifies a putative PERK in lower eukaryotes

**Figure 3:**
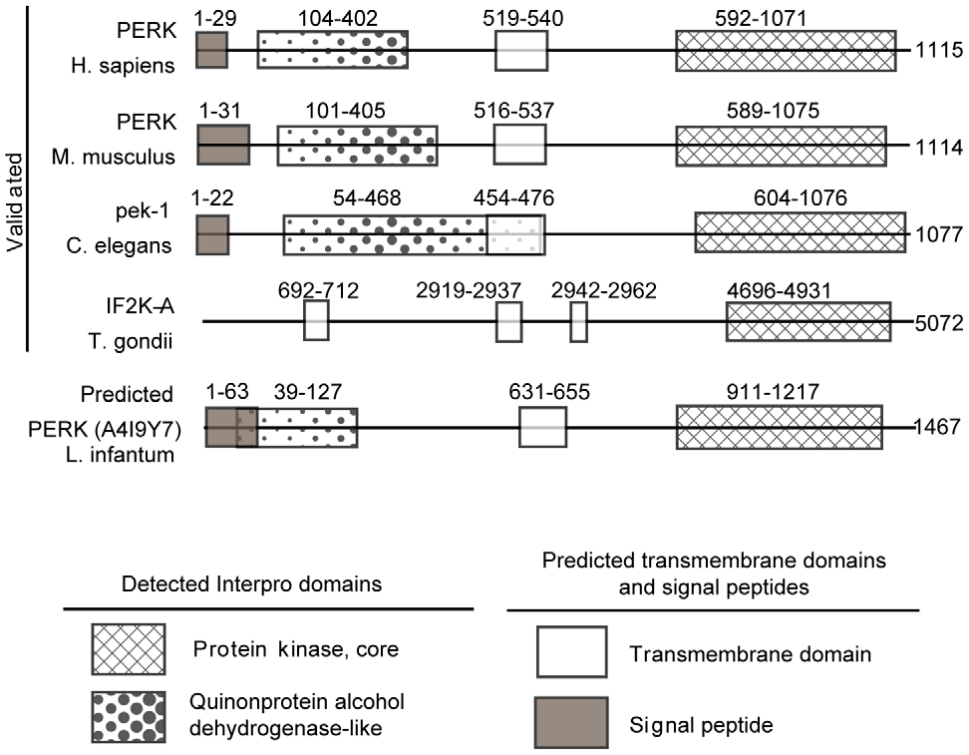
PERK domain structure. The domain structure of the PERK-like protein identified in *L. infantum* compared to validated PERK proteins in Human, Mouse, *C. elegans* and *T. gondii.* The legend describing the protein domains is below.

Because we did not see an absence of domains required for the PERK pathway and there has been evidence of this pathway in *T. gondi* [11], we developed a naïve Bayes*'* classifier to search for PERK in *L. donovani* (and other missing proteins, see Methods). We were able to identify several putative PERK proteins. We then aligned the kinase domains of these proteins to known eIF2α kinase structures in the Protein Data Bank (PDB) [18] (see Methods) using PSI-BLAST [19] to arrive at a single protein in *L. infantum*, *T. cruzi*, and *T. brucei*, the latter of which has been characterized in [15] (Table S2). Consistent with previous findings in *T. gondii* [11], we identified two such proteins in *P. falciparum*, although none were identified in *D. discoideum*. Figure 3 shows the domain structure of the PERK identified in *L. infantum* compared with the domain structure of validated PERK orthologs in human, mouse, *C. elegans* and *T. gondii.* The full alignment of *L. infantum* PERK with other known and putative PERK molecules is depicted in Figure S2.

### PERK-eIF2α pathway is conserved among trypanosomatids

**Figure 4:**
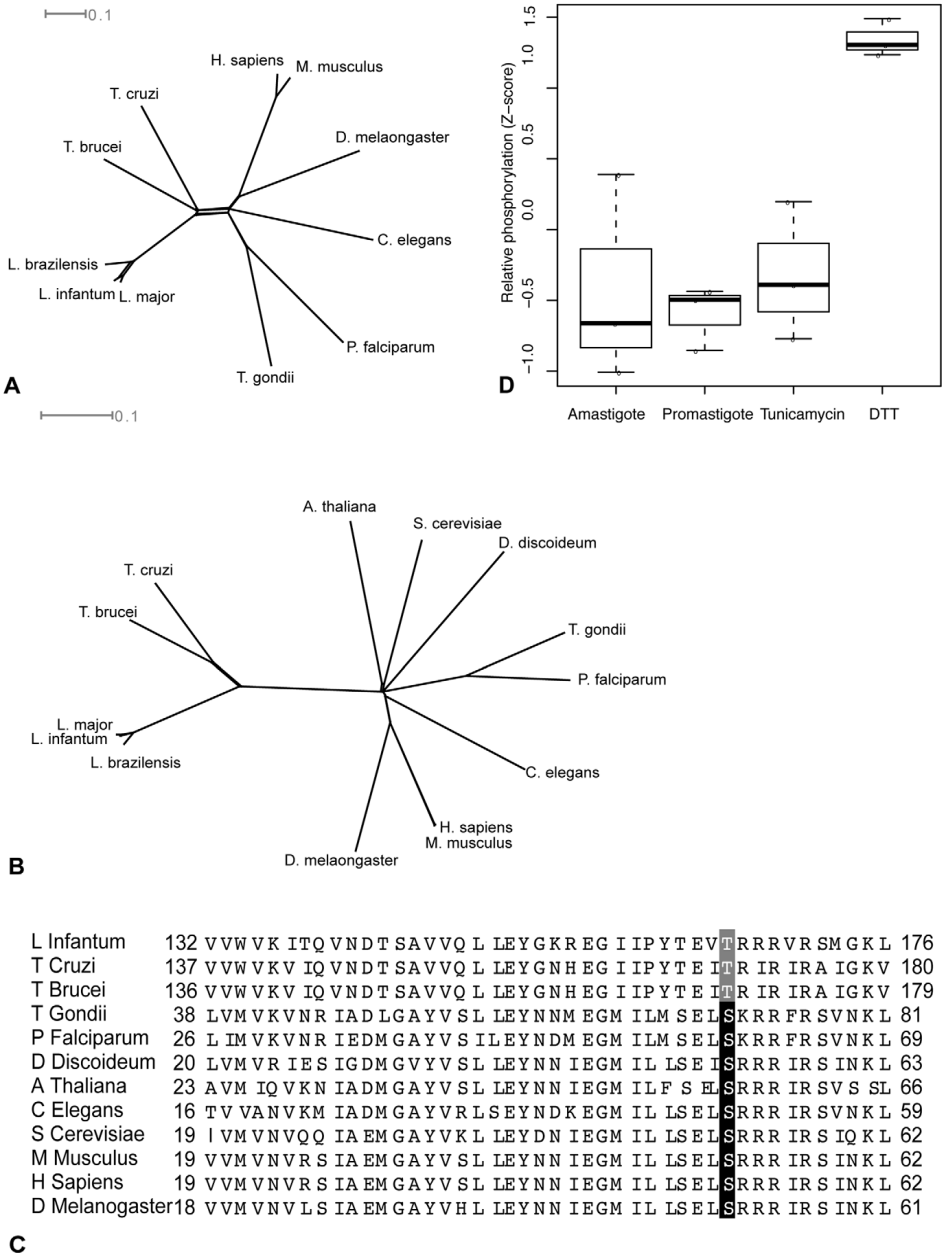
Evolution of PERK pathway. A) Sequence alignment (see Methods) of PERK kinase domains and B) eIF2α phosphorylation sites across eukaryotes. C) Alignment of phosphorylation site of eIF2α across the 12 Eukaryotes in this study. Phosphorylated serine is highlighted in black, while the phosphorylated threonine is highlighted in grey. D) Relative values of phosphorylated threonine in untreated amastigotes (first column), untreated promastigotes (second column) and promastigotes treated with tunicamycin (third column) and DTT (fourth column). Values represent the Z-score of each treatment for each of the three biological replicates.

To investigate whether the putative PERK protein identified in *Leishmania* is capable of phosphorylating eIF2α under ER stress, we performed phylogenetic analysis of both the cytosolic kinase domain of PERK and the phosphorylation site of eIF2α with those of the other species in our study. The phylogenetic trees of the PERK kinase domain (depicted in Figure 4A) and the eIF2α phosphorylation site (depicted in Figure 4B) suggest a divergence in phosphorylation activity in trypanosomatids (which occur on the left of both phylogenetic trees).

This divergence appears to be due to a change in the highly conserved phosphorylation site of eIF2α, depicted in Figure 4C. In all eukaryotes including protozoa such as Apicomplexa, eIF2α is phosphorylated at Ser_51_ (highlighted in black) which is surrounded by a highly conserved motif [20]. However, when eukaryotic phosphorylation sites are aligned with the putative *L. infantum* phosphorylation site, there is a threonine (highlighted in grey) in place of the serine (Figure 4C) suggesting that Thr_166_ is phosphorylated in this species. Additionally, the conserved amino acids in close proximity to Thr_166_ differ from higher eukaryotes; leucine and methionine upstream of Thr_166_ are replaced by proline and tyrosine and the leucine downstream of Thr_166_ has been replaced by valine. Lastly, while eIF2α contains ∼340 amino acids in most eukaryotes, copies of the protein identified in three *Leishmania* species were each over 400 amino acids in length.

Despite this divergence, recent studies in both *T. brucei* [15] and *L. donovani* [21] reported that eIF2α is phosphorylated at Thr_169_ and Thr_166_ in both species respectively. This phosphorylation was shown to decrease protein translation in both organisms [15], [21].

### BiP protein levels in L. donovani do not change in response to UPR stress

While the absence of transcriptional control is not uncommon among trypanosomatids [13], [22], recent evidence of a UPR mediated via post-transcriptional mRNA regulation in *T. brucei* [14] raised the question of whether or not *Leishmania* species could mount a UPR at the protein level despite the absence of transcriptional regulation. To investigate this, we examined BiP protein levels in response to treatment by tunicamycin and dithiothreitol (DTT), two compounds commonly used to induce the UPR [1]. BiP is known to be highly up-regulated by the UPR transcriptional response across metazoan and plants [23] and up-regulated post-transcriptionally in *T. brucei* [14]. We included tubulin and A2 proteins as controls. While the precise role of A2 is unknown, it is present in low levels in cultured promastigotes and has been shown to be expressed in response to cellular stress, such as increased temperature, as well as in response to stimulation of differentiation [24], [25], [26].

**Figure 5:**
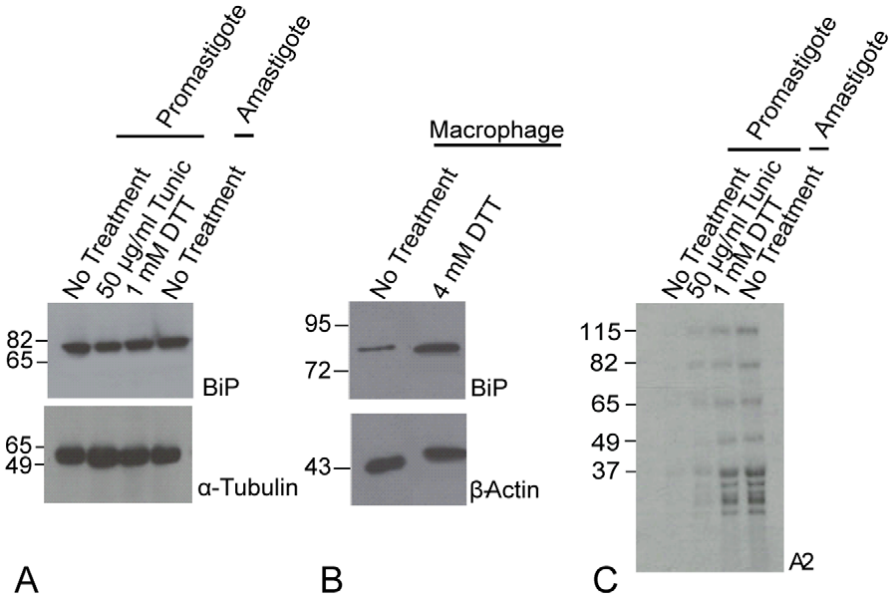
Western blot analysis. Western blot analysis of BiP and A2 protein expression in promastigote and amastigote cultures treated for 8 hours with UPR inducing agents, tunicamycin and DTT. The same *L. donovani* cell lysates were used for the BiP, A2 and α-tubulin blots. A) The levels of BiP protein expression is not affected by the presence of either tunicamycin (lane 2) or DTT (lane 3) when compared to untreated promastigotes (lane 1) or amastigotes (lane 4). B) BiP protein levels in host macrophages (top panel) with β-actin as a control (bottom panel) C) The A2 protein family expression in promastigotes treated with tunicamycin of DTT and in amastigotes as indicated.

As shown in the Western blot in Figure 5A, BiP and tubulin protein levels in *L. donovani* did not change in response to treatment with tunicamycin or DTT. As expected, BiP protein levels in host cell macrophages increased in response to DTT treatment (Figure 5B). In comparison, DTT induced expression of the A2 family of proteins in promastigotes to levels similar to those observed in heat differentiated axenic amastigotes indicating that the chemical is causing stress to the organism (Figure 5C). The weak induction of A2 proteins by tunicamycin indicates that this chemical does not cause high levels of stress in cultured *L. donovani* promastigotes. A2 proteins migrate as a ladder in SDS-PAGE since it is a multigene family in *L. donovani* where each member has a different number of a ten amino acid repeat sequences [24]. Because *L. donovani* cultures failed to proliferate at the concentrations of DTT used to induce a change in BiP protein expression in *T. brucei* [14] (shown in Figure S3, Panel A) A2 served as a way to illustrate that DTT was inducing a stress response in *L. donovani*. The results demonstrate that DTT induced a stress response in *L. donovani,* as determined by increased A2 protein expression, but this did not result in the induction of BiP.

### Phosphorylation of eIF2α upon UPR induction provides evidence of translational control originating from the ER in Leishmania

To assess whether ER stress activates eIF2α phosphorylation in *L. donovani*, we used nano-LC-multiple reaction monitoring (MRM)-MS ([21], see Methods) to measure eIF2α phosphorylation levels in promastigotes exposed to DTT and tunicamycin. In this method, an aliquot of a heavy phosphopeptide isotope was used to identify and quantitate the level of phosphorylated Thr_166_ in *L. donovani* promastigotes (see Methods). This experiment revealed increased phosphorylation of eIF2α upon DTT treatment (Figure 4D). Together with the high degree of similarity with the *T. brucei* PERK-eIF2α pathway [15] and direct evidence that this phosphorylation attenuates translation in *L. donovani* [21], this increased phosphorylation illustrates that ER stress activates a PERK-eIF2α associated translational attenuation pathway in these organisms.

### Induction of the UPR by DTT leads to reduced viability of Leishmania donovani

**Figure 6:**
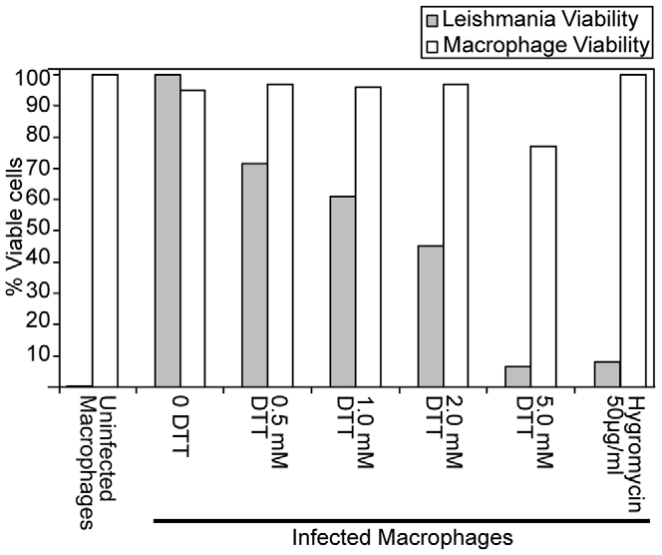
Treatment of *L. donovani* infected macrophages with DTT. Macrophages were infected for 12 hr and parasite load along with macrophage viability were determined 24 hours later as detailed in Methods. Figure depicts a single representative experiment of three replicates. Lane1: Non-infected macrophage cells. Lane 2: No-treatment control. Lane 3–6: post-treatment where increasing concentrations of DTT were added for 24 hrs after infection and then parasite load and macrophage viability were determined. Lane 7: post treatment with 50 µg ml^−1^ of hygromycin.

To determine if the UPR in *Leishmania* is sufficient to protect the organism from ER stress, we treated *Leishmania* within host macrophages with DTT and then measured the viability of both the macrophage cell and the intra-macrophage amastigote. As shown in Figure 6, intracellular amastigotes were more sensitive to DTT than the host macrophage, suggesting that the translational response present in *Leishmania* is not as effective at protecting the parasite as the UPR present in metazoa. These results were confirmed in axenic cultures of *L. donovani* promastigotes (Figure S3A) that were also significantly more sensitive to DTT than were uninfected macrophages (Figure S3B).

## Discussion

The UPR is an important set of signaling pathways that protects cells from pharmacological and environmental insults that affect the performance of the ER. Studying the evolution of the individual pathways within the ER can shed light on their importance in disease. However, the inability of bioinformatic tools such as BLAST [27] to identify known orthologs in the UPR (e.g. Xbp1/Hac1) have made it difficult to characterize the UPR in organisms more ancient than yeast such as *Leishmania*. The identification of Atf6 in plant [10] and PERK in Apicomplexa [11] failed to resolve the current models of pathway evolution proposing that the Ire1 transcriptional pathway predates the Atf6 and PERK pathways [8], [9]. The work presented here provides a more comprehensive view of UPR evolution.

The UPR was discovered in yeast as a transcriptional response to the accumulation of misfolded proteins in the ER and has since been identified in metazoa and plant [8]. Given the divergent transcriptional machinery in protozoa [13], [22], the absence of a transcriptional response to misfolded proteins in organisms such as *G. lamblia* [12] and *T. brucei* [13] is not surprising. Our model of protein domains in the UPR (Figure 2) shows that this absence occurs throughout protozoan parasites. However, the recent identification of a UPR-like response at the protein level through mRNA regulation [14] suggests that ER chaperones such as BiP could still be up-regulated at the protein level in response to ER stress. We illustrated in Figure 5 that this is not the case in *L. donovani*, warranting further exploration of this mechanism in other organisms.

Evidence of translational attenuation in response to ER stress was first identified in *C. elegans* [8] and later in metazoa. In yeast and plant, translational attenuation is activated in a similar manner but by a cytosolic protein, Gcn2, thus making it independent of ER stress [8]. In *T. gondii*, activation of a PERK ortholog via disassociation with BiP upon ER stress was shown to decrease protein translation, suggesting that this pathway is more ancient than previously believed [11]. While our domain-based identification of a PERK ortholog in *L. donovani* (Figure 3A) suggested that this pathway was also conserved in *Leishmania* species, localization of the *T. brucei* PERK ortholog to the flagellar pocket [15] suggested that PERK in *L. donovani*, given its high degree of similarity with its trypanosome orthologs (Figures 4) may behave in a similar manner. However, our experiments illustrate that eIF2α is specifically phosphorylated when ER stress is induced (Figure 4D). Evidence of this phosphorylation activity decreasing overall translation in both *T. brucei* [15] and *L. donovani* [21] suggests a direct tie between ER stress and general translation levels in *L. donovani*.

The existence of translational control in both *Leishmania* and *Toxoplasma* is surprising given its absence in other organisms such as yeast and plant. Mathematical models of the UPR pathways [28] suggest that the PERK pathway is vital for cells with more secretory activity such as specialized secretory cells in metazoa. In parasites, protein secretion may be crucial to allow the parasite to survive in diverse environments including the mammalian host [29]. Thus, for an organism like *L. donovani* that relies on secretion for survival and has little transcriptional control, it is logical that the UPR involves the PERK pathway without transcriptional control.

This work does not address the evolution of mRNA regulation in response to ER stress. In metazoa, the ability of Ire1 to mediate mRNA decay of specific transcripts, termed Ire1-dependent decay (RIDD) [30] characterizes a third mechanism by which cellular protein levels are modified via ER stress. In *T. brucei*, mRNA regulation of transcripts is responsible for up-regulating a number of ER proteins at the protein level including BiP at very high concentrations of DTT [14]. While the absence of differential expression of BiP at the protein level (Figure 5) suggests that mRNA regulation does not play a similar role in *Leishmania*, the evolution of this mechanism should be further explored.

Lastly, our observation that *L. donovani* was more sensitive to DTT treatment than host macrophages (Figure 6) indicates that the UPR may be exploited to weaken the organism as it infects the host. Though there exist drugs to treat leishmaniasis infection [31], possible therapies that combine stimulation of the UPR response in *Leishmania* with leishmanicidal drugs are of considerable interest due to the rapid evolution of these organisms. Tunicamycin does not have such a detrimental effect, as previous studies have shown that treatment with this chemical does not cause N-glycosylated proteins to be retained in the ER [32] and therefore would not cause ER stress. For this reason we saw no phosphorylation of eIF2α upon tunicamycin treatment.

In conclusion, we used targeted computational techniques to characterize the evolution of the UPR in eukaryotes. We provided evidence for the existence of a PERK translational control pathway in *L. donovani* as well as an absence of UPR mediated transcriptional or post-transcriptional control. This alternate UPR in *Leishmania* makes it more sensitive to ER stress, providing a novel approach to drug development that can be exploited in other parasites that possess a similar UPR.

## Materials and Methods

### Identification of putative orthologs from sequence data

We collected a list of human, yeast and plant proteins involved in UPR signaling from various reviews and literature sources [1], [8], [9]. We ran InParanoid [33] using these sequences (from all three species) against the protozoan genomes listed in Table 1 using first the default parameters and then lower bit-score cutoffs of 40 and 30 with the BLOSUM 45 matrix to lower the degree of expected conservation between matches. We also used OrthoMCL [34] to search for well-conserved protein orthologs, and BLAST [27] (default parameters) to search for protein (blastp) and DNA (tblastn) sequences with a partial and/or weaker conservation. We curated the results of these searches to remove spurious orthologs.

For PERK and Atf6, where alternate forms of the protein were identified outside of mammals, we used *T. gondii* PERK [11] and *A. thaliana* Atf6 (AtbZIP60) [10] sequences to seed our search for these proteins. BLAST was able to identify orthologs of *T. gondii* PERK in *P. falciparum*.

### Protein domain model of UPR evolution

We collected the domains in each UPR protein identified in Table S1. For each domain *d*, we calculated the ratio of the likelihood of observing *d* in a UPR-related protein *p*, 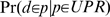 over the likelihood of observing a non-UPR protein *p* with domain *d* in the organism 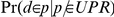. We then performed hierarchical clustering (Ward*'*s algorithm, Euclidean distance) and plotted in R/Bioconductor (http://www.bioconductor.org) (Figure 2). Exact specificity measurements are in Table S3.

### Naïve Bayes' classifier

We collected the domains for each of the proteins in Table S1. For each protein that was missing orthologs, we constructed a naïve Bayes*'* classifier that gives a score to each functional domain *d* that is present in the Interpro database [17]. The classifier scores each domain *d* with a score of 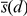 which represents the mean value of 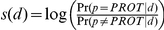 as measured in all species in which protein of interest PROT has been identified. *s(d)* represents posterior log-odds ratio of a protein being protein PROT given the presence of domain *d* in a particular species *s*. If a domain *d* is not found in protein PROT, then *s(d)* = 0. For a particular protein *p* with domain set D, we calculated the total log likelihood that *p* is an ortholog as: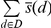. The high-scoring results for each PERK, along with additional curation described in Methods, are in Table S2. We searched for orthologs of Ire1, Atf6, Xbp1 and DNAJC3 but found none.

### Curation and structural alignment

We ran PSI-BLAST [19] across the high-scoring PERK proteins (results in Table S2) to identify those whose kinase domain most closely resembled the eIF2 kinase domain structure available in PDB: 2a19/2a1a [35] and 1zy4/1zyD/1zyC [36]. Through this search, we were able to identify a putative PERK in *L. Infantum* that shares close homology with other trypanosomatid PERK proteins. We were also able to identify two proteins in *P. falciparum* and no proteins in *D. discoideum* whose best PDB structure hit was one of the eIF2 kinase domains. Lastly, we collected transmembrane domain predictions for the final PERK candidates from a number of sources to account for the fact that no single predictive tool is perfect [37]. All candidate transmembrane domains are depicted in Figure S1. The full alignment of putative and validated PERK proteins is depicted in Figure S2. The final proteins are listed in Table S1.

### Parasite Cultures

The *Leishmania donovani* 1S/Cl2D promastigotes were routinely cultured as previously described [38]. *L. donovani* promastigotes were induced to differentiate into axenic amastigotes by incubation overnight in amastigote culture medium (37°C, pH 5.5 in RPMI 1640 plus 25% fetal bovine serum, [39]). Tunicamycin (1–100 µg ml^−1^) and DTT (0.1 mM–10 mM) were added to the growth medium. The *L. donovani* 1S2D [40] engineered to express an ectopic luciferase gene (provided by Dr. Martin Olivier) as a marker for viability was cultured in *Leishmania* media [38] supplemented with 38 µg/ml of G418. Luciferase activity was determined in either *L. donovani* or *L. donovani-*infected macrophage cells as previously described [40].

### Western blot analysis

Promastigote cultures, tunicamycin or DTT-treated promastigote cultures and amastigotes were washed two times with chilled PBS, re-suspended to 5.0×10^6^cells/10 µl, and immediately lysed with boiling 2×SDS-PAGE sample buffer, as previously described in [41]. Detection of A2 proteins was performed as described previously with the anti-A2 monoclonal antibody [24]. For the BiP detection, anti-BiP antibodies kindly provided by Dr. J. Bangs, were used in a 1∶1000 dilution and the secondary antibody was donkey anti-rabbit IgG (Amersham). To insure equal loading of protein in each lane, and as a negative control for the UPR, cells were also blotted with anti-tubulin antibodies (Oncogene). The same *L. donovani* cell lysates were used for the BiP, A2, and tubulin Western blots.

### Determination of UPR-induced eIF2α phosphorylation

Logarithmic phase *L. donovani* promastigotes (4.4×10^7^ cells/ml) were treated with 0.5 mM DTT or 50 µg ml^−1^ tunicamycin for eight hours as described above. Following treatment, 2×10^9^ cells were collected, washed three times with ice cold PBS supplemented with phosphatase inhibitors (1 mM Sodium orthovanadate (Na_3_VO_4_), 50 mM NaF and 5 mM beta-glycerophosphate), and divided into two aliquots of 1×10^9^ cells. Cell pellets were then lysed using a buffer containing 1% w/v sodium deoxycholate, 25 mM ammonium bicarbonate, and three phosphatase inhibitors (5 mM NaF, 5 mM Na_3_VO_4_, and 10 mM β-glycerophosphate). One mg of protein from each sample was reduced with DTT, and cysteine sulfhydryls alkylated with iodoacetamide and then subjected to trypsin (20 ug) digestion for 16 h at 37°C.

A heavy version of the EGIIPYTEV(pT)R phosphopeptide (+10 Da) was spiked into samples and the resulting peptide mixes were mixed with TiO_2_ beads and phosphopeptides eluted in two steps, using 30 and 50% ACN in 0.5% NH_4_OH. The enriched phosphopeptides were subjected to nano-LC-multiple reaction monitoring (MRM)-MS analysis at the Genome BC Proteomics Centre at the University of Victoria [42]. All data was analyzed using MultiQuant 1.1 (Applied Biosystems). The ratio of endogenous EGIIPYTEV(pT)R phosphopeptide levels in the samples to those of the heavy phosphopeptide (averaged from five MRM transitions) is then normalized to a Z-score across all conditions for each of the three samples and reported in Figure 4D.

### Macrophage infection with L. donovani and treatment with DTT

Murine macrophages derived from raw 264.7 (ATCC TIB-71) cells (1×10^5^ ml^−1^/well) were infected with stationary phase *L. donovani* promastigotes containingan ectopic firefly luciferase gene in a 20∶1 ratio of parasite to macrophage cells for 12 hrs. Free extracellular parasites were washed away from the adherent macrophages and cells were treated with either DTT concentrations ranging from 0.5–5 mM or with 50 µg ml^−1^ hygromicin for 24 h. Treated infected macrohages were then harvested and macrophage viability was assessed by the AlamarBlue ® bioassay (Invitrogen) and parasite viability determined by measuring luciferase activity.

## Supporting Information

Figure S1 Full domain characterization of putative PERK proteins in metazoa, Apicomplexa and trypanosomatids. We used a combination of tools to predict transmembrane domains and signal peptides as described in the Methods to account for differences between prediction tools. The legend describing the protein domains is on the right-hand side.
(PDF)

Figure S2 Results of ClustalW alignment of putative PERK proteins from each species evaluated in this study (plus two additional *Leishmania* species). Rows are described by species and, in parenthesis, protein identifier for each putative PERK protein. Due to the excessive size of the *Toxoplasma gondii* PERK the first part of this protein (amino acids 1–3276) was removed. Jalview (www.jalview.org) was used to visualize the alignment. Numbers on either side of the sequence indicate the position in the protein, and coloring indicates degree of sequence conservation where darker purple reflects more highly conserved amino acids.
(PDF)

Figure S3 (A) Proliferation and viability analysis of *L. donovani* promastigotes in the presence of DTT. (B) Proliferation and viability analysis of macrophages in the presence of DTT.
(PDF)

Table S1 Protein identifiers of the UPR proteins in the 12 species in this study. For each protein family (indicated by a row describing the family name in boldface), each row indicates a different species identifier. In families for which there are multiple paralogs in a single species, (e.g. Atf6) paralogous genes are replicated in the columns. Absent entries indicate that no ortholog was found for that particular species and protein family.
(XLS)

Table S2 Putative PERK orthologs identified by the Naïve bayes*'* classifier. Column 1 indicates the species in which the protein was identified, column 2 indicates the Uniprot identifier, column 3 indicates the log-likelihood score and column 4 indicates the domains present on the protein. Columns 5 and 6 indicate the PSI-BLAST E-values against the known eIF2α kinase structures in PDB.
(XLS)

Table S3 UPR specificity scores of each protein domain in each species.
(XLS)
