# Supplementary figures and images for: Intracellular Eukaryotic Parasites Have a Distinct Unfolded Protein Response
Source: PLoS One. 2011 Apr 29;6(4):e19118. doi: 10.1371/journal.pone.0019118 (PMC3084755; doi:10.1371/journal.pone.0019118)

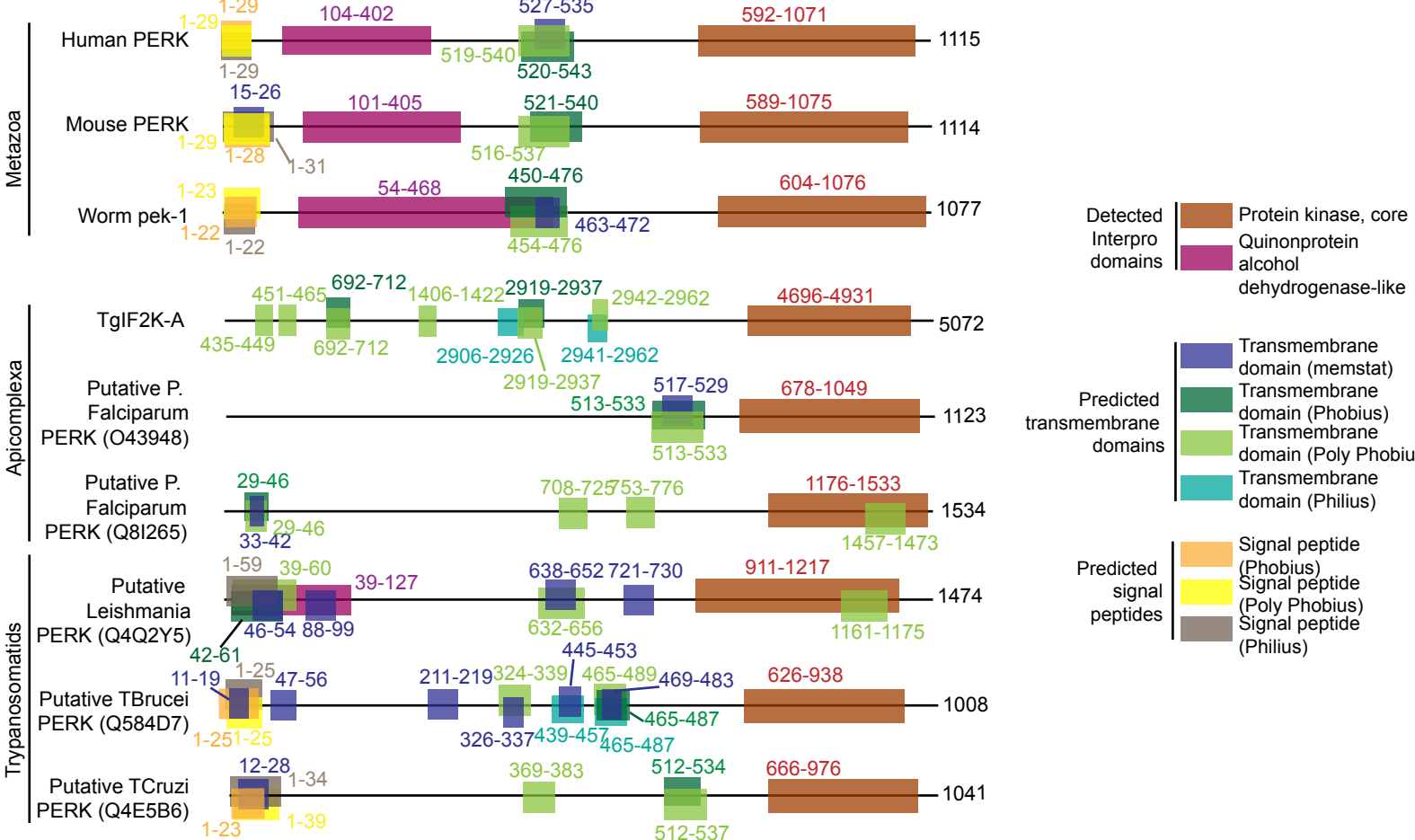

A

Supplement: Figure S1 — Full domain characterization of putative PERK proteins in metazoa, Apicomplexa and trypanosomatids. We used a combination of tools to predict transmembrane domains and signal peptides as described in the Methods to account for differences between prediction tools. The legend describing the protein domains is on the right-hand side. (PDF) [file pone.0019118.s001.pdf]

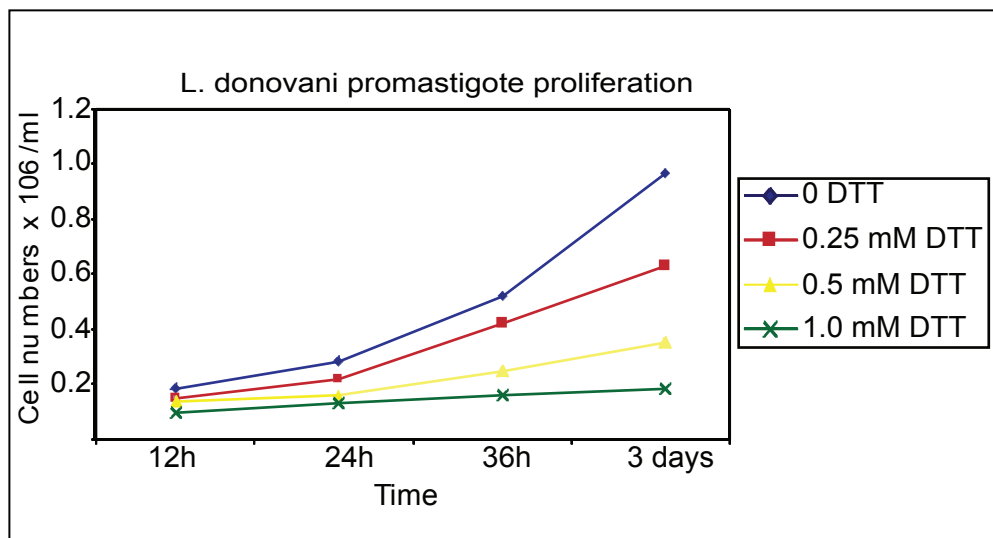

A

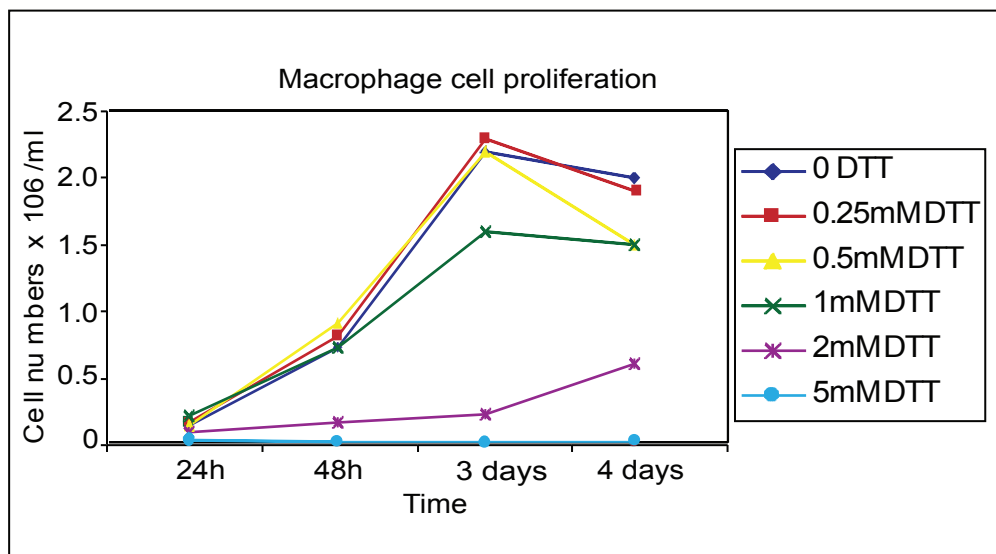

B

Supplement: Figure S3 — (A) Proliferation and viability analysis of L. donovani promastigotes in the presence of DTT. (B) Proliferation and viability analysis of macrophages in the presence of DTT. (PDF) [file pone.0019118.s003.pdf]
