# Supplementary material for: Intracellular Eukaryotic Parasites Have a Distinct Unfolded Protein Response
Source: PLoS One. 2011 Apr 29;6(4):e19118. doi: 10.1371/journal.pone.0019118 (PMC3084755; doi:10.1371/journal.pone.0019118)
Supplement: Figure S2 — Results of ClustalW alignment of putative PERK proteins from each species evaluated in this study (plus two additional Leishmania species). Rows are described by species and, in parenthesis, protein identifier for each putative PERK protein. Due to the excessive size of the Toxoplasma gondii PERK the first part of this protein (amino acids 1–3276) was removed. Jalview (www.jalview.org) was used to visualize the alignment. Numbers on either side of the sequence indicate the position in the protein, and coloring indicates degree of sequence conservation where darker purple reflects more highly conserved amino acids. (PDF) [file pone.0019118.s002.pdf]

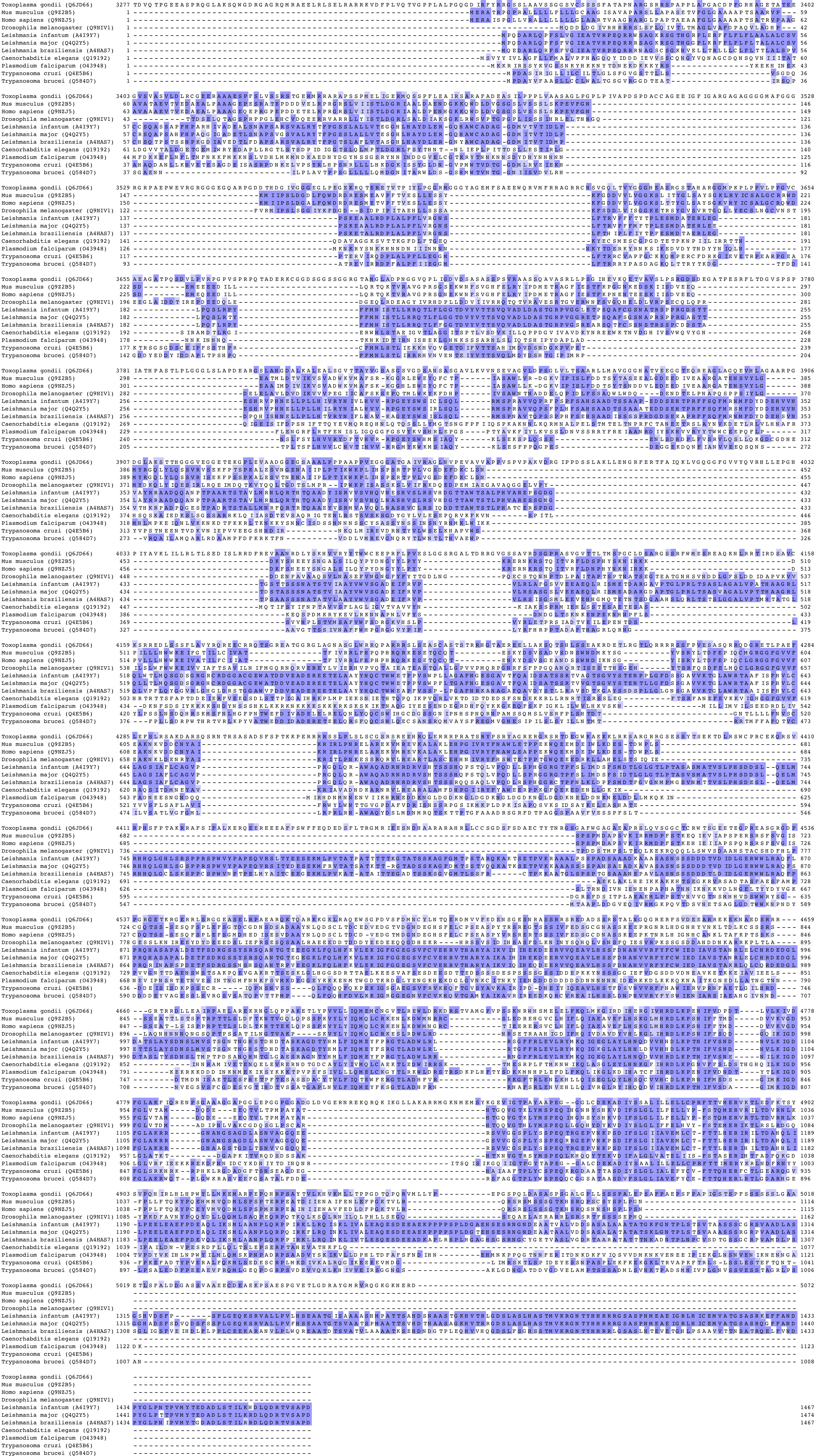
Toxoplasma gondii (Q6JD66)
Mus musculus (Q9Z2B5)
Homo sapiens (Q9NZJ5)
Drosophila melanogaster (Q9NIV1)
Leishmania infantum (A4I9Y7)
Leishmania major (Q4Q2Y5)
Leishmania braziliensis (A4HAS7)
Caenorhabditis elegans (Q19192)
Plasmodium falciparum (O43948)
Trypanosoma cruzi (Q4E5B6)
Trypanosoma brucei (Q584D7)
